# Effects of Diazinon Exposure on Urinary Shedding of *Leptospira interrogans* Serogroup Hebdomadis in Mice

**DOI:** 10.3390/toxics11040361

**Published:** 2023-04-11

**Authors:** So Shinya, Devinda S. Muthusinghe, Nobuo Koizumi, Kumiko Yoshimatsu, Shouta M. M. Nakayama, Mayumi Ishizuka, Yoshinori Ikenaka

**Affiliations:** 1 Laboratory of Toxicology, Graduate School of Veterinary Medicine, Hokkaido University, Sapporo 060-0818, Japan; 2 Institute of Genetic Medicine, Hokkaido University, Sapporo 060-0808, Japan; 3 Department of Bacteriology I, National Institute of Infectious Diseases, Shinjuku, Tokyo 162-8640, Japan; 4 Biomedical Science Department, School of Veterinary Medicine, The University of Zambia, Lusaka 32379, Zambia; 5 Translational Research Unit, Veterinary Teaching Hospital, Faculty of Veterinary Medicine, Hokkaido University, M18, W9, Kita-ku, Sapporo 060-0818, Japan; 6 One Health Research Center, Hokkaido University, M18, W9, Kita-ku, Sapporo 060-0818, Japan; 7 Water Research Group, Unit for Environmental Sciences and Management, North-West University, Potchefstroom 2520, South Africa

**Keywords:** immunotoxicity, insecticide, pesticides, pollutant, infectious disease

## Abstract

Wild rodents are natural hosts of *Leptospira* spp. and are exposed to various pesticides, some of which are immunotoxic. Rodent urine is an important source of infection for humans and other animals. We evaluated the effects of pesticide exposure on *Leptospira* growth in mice. Diazinon, at doses of 0.2, 1, and 5 mg/kg/day, was orally administered continuously to mice infected with *Leptospira interrogans* serogroup Hebdomadis for 32 days. The numbers of *L*. *interrogans* in urine and kidney tissues were significantly lower in mice exposed to 5 mg/kg/day diazinon than in unexposed mice (*p* < 0.05). The urinary concentration of 2-isopropyl-6-methyl-4-pyrimidinol, the metabolite of diazinon, was comparable with the concentration at which viability of *L. interrogans* was decreased in in vitro assay, suggesting that it had toxic effects on *L*. *interrogans* in the proximal renal tubules. Diazinon exposure reinforced *Leptospira*-induced expression of inflammatory cytokine genes in kidney tissues, and an enhanced immune system might suppress *Leptospira* growth. These results suggest that diazinon exposure may not increase the risk of *Leptospira* transmission to humans through mice. This novel study evaluated the relationship between pesticide exposure and *Leptospira* infection in mice, and the results could be useful for risk assessment of leptospirosis.

## 1. Introduction

Leptospirosis is a zoonotic disease that causes a variety of symptoms in humans and susceptible animal species [1,2]. *Leptospira* spp. colonize the kidney tissues and are excreted in the urine of reservoir animals such as rodents. Transmission occurs through direct contact with the urine of reservoir animals or indirectly via contact with the environment, such as soil and water, contaminated with their urine [2]. Thus, the urine of wild rodents is an important infectious source of *Leptospira* spp. for humans or other animals.

Characterizing the urinary shedding of *Leptospira* spp. in rodents is a useful step in the risk assessment of human leptospirosis [3,4]. In natural environments, rodents are constantly exposed to various pesticides, including insecticides [5,6,7]. However, the effects of pesticide exposure on urinary shedding of *Leptospira* spp. by rodents have not been investigated. 

Diazinon (DZN) is one of the most widely used insecticides; it is applied to various kinds of crops (e.g., corn, turnip, and onion) at doses of approximately 15–45 g/ha and is detected in environments globally [8,9,10]. DZN elicits varying immune system effects on exposed animals depending on the exposure levels. At levels of 25–50 mg/kg/day, immunosuppression has been reported in mice; in the spleen, decreased weight, decreased cytokine gene expression levels of interleukin-2 (IL-2), IL-4, IL-10, and interferon-γ (IFN-γ), and suppressed humoral and cellular immune responses have been observed. In addition, decreased B cell concentrations in blood and suppression of lymphocyte proliferative responses have been reported [11,12]. In contrast, increased mRNA expression levels of cytokine genes, including tumor necrosis factor-α (TNF-α) and IL-10 in blood, have been observed in response to lower concentrations (1.5–6.5 mg/kg/day) of DZN exposure; this may indicate enhanced immune responses [13,14]. These cytokines are involved in the immune response to leptospirosis, and the DZN-induced effects may affect the growth of *Leptospira* spp. in infected mice [15,16,17].

At more than 30 mg/kg/day, DZN causes histological changes in the kidneys, including degeneration and loss of glomerular integrity [18]. If histological changes also occur at the concentration equivalent to the environmental exposure level, urinary shedding of *Leptospira* spp. could be affected. In addition, DZN could show lethal effects on *Leptospira* spp. in the kidney, although there is no report regarding DZN-induced toxicities for the bacteria.

The purpose of this study was to evaluate the effect of DZN exposure on urinary shedding of *Leptospira* spp. in mice, considering the environmental situation in which rodents are exposed to both *Leptospira* spp. and DZN. The number of *L. interrogans* in urine and kidney tissue was investigated following oral administration of DZN to mice infected with *L. interrogans*. Additionally, cytokine gene expression levels and histological changes in the kidneys were determined following exposure.

## 2. Materials and Methods

### 2.1. Culture Conditions of L. interrogans

*Leptospira interrogans* serogroup Hebdomadis strain OP84 [19] was used in this study. The strain was stored in Korthof’s medium containing 10% glycerol at −80 °C until each experiment. For the in vitro viability assay, the strain was thawed and cultured in liquid Korthof’s medium at room temperature at first passage and then cultured in Ellinghausen–McCullough–Johnson–Harris (EMJH) medium after second passage. In vitro passage was conducted fewer than three times before the assay. For in vivo infection, the strain was thawed and passaged twice using liquid Korthof’s medium and then inoculated into mice.

### 2.2. In Vitro Viability Assay of L. interrogans

The lethal dose of DZN (Fujifilm, Tokyo, Japan) and its major metabolite, 2-isopropyl-6-methyl-4-pyrimidinol (IMP) (Santa Cruz Biotechnology, Santa Cruz, CA, USA), were investigated according to the reported method [20]. In a round bottom 96-well plate, 180 µL of 5 × 10^6^ cell/mL 7 day-cultured OP84 strain was inoculated into each well. The number of leptospiral cells was determined using a Thoma cell counting chamber (Yazawa Science, Tokyo, Japan) and dark field microscopy. Then, 20 µL of serial two-fold diluted DZN, IMP, or ampicillin (as a positive control) in EMJH medium containing 1% methanol ranging from 0.03 to 500 ppm was added. Each plate also included a negative control (EMJH containing 1% methanol without DZN or IMP). After 3 days of incubation at room temperature, 20 µL of 10-fold diluted AlamarBlue Cell Viability Reagent (Thermo Fisher Scientific, Waltham, MA, USA) was added to each well. After an additional 2 days of incubation, absorbance at 570 nm was measured using a spectrometer (Multiskan GO, Thermo Fisher Scientific). Cell viability was represented as a ratio with the negative control wells. Assays were performed in triplicate.

To confirm the stability of DZN and IMP in EMJH medium during the assay, wells containing *L. interrogans* and four concentrations of DZN or IMP at 1.95, 7.8, 15.6, and 250 ppm were prepared; 100 µL of medium was collected before and after the assay and the concentration of DZN and IMP was measured as described below (Section 2.10).

### 2.3. Leptospira Infection and Exposure to DZN

Seven-week-old male ICR mice (*n* = 72) were used for the in vivo experiments. Mice were purchased from CLEA (Tokyo, Japan) and kept at 20–23 °C under a 12 h/12 h light/dark cycle. Water and food (CE-2; CLEA) were given ad libitum. Experiments were performed after a 2 week acclimation period.

Mice were categorized into eight groups (each group: *n* = 9): control; low, medium, and high-dose DZN exposure (LD, MD, HD, respectively); *Leptospira* infection without DZN exposure (Lepto); and *Leptospira* infection with low, medium, and high-dose DZN exposure (Lepto + LD, Lepto + MD, and Lepto + HD, respectively). 

The mice in all four groups infected with *Leptospira* were intraperitoneally inoculated with 3 × 10^6^ cells of strain OP84 in the log phase suspended in 150 µL of Korthof’s medium and then kept in mesh cages for approximately 1 h. The number of leptospiral cells was determined as mentioned above. The same volume of Korthof’s medium was intraperitoneally inoculated to all other groups.

Following inoculation, excreted urine was collected in 1.5 mL tubes at 6, 8, 10, 13, 16, 18, 21, 24, 27, and 31 days post infection (dpi). Urine samples were kept at −80 °C until analysis. For DZN exposure, at 31 dpi, DZN was dissolved in the drinking water of all the groups except the control and Lepto groups. DZN was then provided orally to the mice ad libitum. The amount of water that the mice drank was estimated as 5 mL/day [21]. The dose of DZN used was 0.2, 1.0, and 5.0 mg/kg/day for low, medium, and high doses, respectively. These doses were set based on the estimated maximum exposure dose (6.13 mg/kg/day) as defined by the deposit of DZN at the recommended dosage on grapes (9.04 mg/kg) [22]. With the assumption that mice depended entirely on grapes contaminated with 9.04 mg/kg of DZN for their diet, an estimated exposure dose can be calculated as 6.13 mg/kg/day using the parameters of 25 g of body weight [23], 0.59 kcal/g of energy in grapes (Food Composition Database provided by Ministry of Education, Culture, Sports, Science and Technology [24]), and 10 kcal/day of energy requirements for mice [23]. DZN exposure was continued until 63 dpi.

During the exposure period, urine samples of *Leptospira*-infected mice were collected at 34, 37, 41, 45, 48, 51, 53, 56, and 63 dpi, as described above. All animals survived without any abnormalities during experiments. At 63 dpi, mice were sacrificed by CO_2_ inhalation. After euthanasia, kidney tissues were collected immediately. A fraction of the right renal cortex was stored at −20 °C in 2 mL of RNAlater (Thermo Fisher Scientific) and stored at 4 °C overnight until DNA or RNA extraction. Other fractions of the right renal cortex were stored at −20 °C for quantification of DZN and IMP residue levels. Left kidneys were stored in 10% of formalin for histological examination.

All animal care and experimental procedures were performed in accordance with the Guidelines of the Association for Assessment and Accreditation of Laboratory Animal Care International (AAALAC) and were approved by the Animal Care and Use Committee of the Graduate School of Veterinary Medicine, Hokkaido University (Approval number: 22-23).

### 2.4. DNA Extraction from Kidney and Urine Samples

Genomic DNA was extracted from 25 mg of the renal cortex and 20 µL of urine of *Leptospira*-inoculated mice using QIAamp DNA Mini Kit (Qiagen, Victoria, Australia). The concentrations of extracted DNA from the renal cortex were determined using Nano Drop Spectrometer ND-1000 (Thermo Scientific, Rockford, IL, USA) and adjusted to 50 ng/µL with double distilled water (DDW) for quantitative PCR. DNA from urine samples was applied to quantitative PCR without concentration adjustment, as described by Soupé-Gilbert et al. [25].

### 2.5. RNA Extraction and cDNA Synthesis from Kidney

Total RNA was extracted from approximately 20 mg of the renal cortex using RNA Basic Kit (FastGene, Tokyo, Japan). The concentration of extracted RNA was determined using Nano Drop Spectrometer ND-1000. cDNA synthesis with 500 ng of extracted RNA was conducted using ReverTra Ace qPCR RT Master Mix with gDNA remover (Toyobo, Osaka, Japan). cDNA was diluted to 50 ng/µL with DDW for absolute quantitative PCR.

### 2.6. Real-Time PCR

Real-time PCR was performed using QuantStudio 12K Flex Real-Time PCR System and software version 1.2.2 (Thermo Fisher Scientific). PCR reactions were conducted in 10-µL volumes containing Fast SYBR Green Master Mix (Thermo Fisher Scientific) and 300 nM forward and reverse primers. Sequence information of the primers used in this study are listed in Table S1. The PCR condition was as follows: initial denaturation at 95 °C for 20 s, 40 cycles of denaturation and amplification of target sequences at 95 °C for 1 s, and 60 °C for 20 s, followed by melting curve analysis to confirm the specificity of amplified products. The efficiency of each primer set was calculated using eightfold dilution of the template DNA, and all primer sets showed acceptable efficiencies within 95–105% (Table S1).

### 2.7. Quantification of Leptospiral DNA in Kidney and Urine

Leptospiral DNA in mouse kidney and urine samples was quantified by absolute quantification of 16S rRNA gene. A standard curve was obtained using serially diluted genomic DNA extracted from the OP84 strain. The mouse ribosomal protein L18 (RPL18) gene was simultaneously quantified as a housekeeping gene to validate the quantity and quality of DNA samples. Quantity of leptospiral DNA in kidney and urine samples was shown as leptospiral genome equivalent per μg genome and per ml of urine, respectively, considering a genome size of 4.6 Mbp corresponding to *L. interrogans* Fiocruz L1-130 [26].

### 2.8. Quantification of Expression Levels of Cytokine Genes in Kidney Tissues

mRNA expression levels of cytokine genes including IL-1β, IL-2, IL-4, IL-6, TNF-α, TNF-β, IFN-γ, transforming growth factor-β (TGF-β), macrophage inflammatory protein (MIP-1), and IFN-γ-induced protein-10 (IP-10) were quantified.

Plasmids were constructed to make standard curves for quantifying cDNA copy numbers of targeted cytokine genes. The cytokine genes mentioned above and internal standard candidate genes, including ribosomal protein L 18 (RPL18), glyceraldehyde-3-phosphate dehydrogenase (GAPDH), β-actin, and β-2-microglobulin gene, were amplified from the cDNA libraries prepared above by PCR using SappireAmp Fast PCR Master Mix (Takara, Shiga, Japan). PCR was conducted at 94 °C for 1 min, followed by 35 cycles of amplification of target sequences at 98 °C for 5 s, 60 °C for 5 s, and 72 °C for 5 s, and a final extension at 72 °C for 1 min. The PCR products were purified using QIAquick PCR Purification Kit (Qiagen, Hilden, Germany) and were cloned into pCR2.1-TOPO vector using a TOPO TA Cloning Kit (Thermo Fisher Scientific).

RPL18 gene was used as an internal control because it showed the most stable expression levels between control mice and DZN exposed or *Leptospira*-infected mice among the internal standard candidate genes described above. Expression levels of cytokine genes in each mouse group are represented as a ratio to that of the control group. Ct 35 was applied as the cut-off value for real time PCR analysis.

### 2.9. Histological Examination of Kidney Tissue

Histological examination was performed by Sapporo General Pathology Laboratory (Sapporo, Japan). Kidney tissues (*n* = 3 for each group, total 24 samples) fixed in 10% buffered formalin were embedded in paraffin. The embedded kidney tissues were sectioned and stained with hematoxylin and eosin. Renal cortex tissue was observed using an optical microscope. 

### 2.10. Extraction and Quantification of DZN and IMP in Kidney Tissues, Urine, and EMJH Medium

Five milligrams of renal cortex were added to 500 µL of 1% formic acid in acetonitrile and homogenized with horizontal shaking at a frequency of 30/s for 1 min using Tissue Lyser (QIAGEN, Hilden, Germany). Homogenized samples were centrifuged at 10,000× *g* for 5 min and the supernatant was collected. MonoSpin Phospholipid (GL Sciences, Tokyo, Japan) was preconditioned with 200 µL of 1% formic acid in acetonitrile by centrifugation at 3000× *g* for 5 min. 100 µL of the collected supernatant was passed through the MonoSpin Phospholipid by centrifugation at 3000× *g* for 5 min, and 80 µL of the elute was added to 240 µL of 0.1% formic acid in DDW and transferred to a plastic vial for quantitative analysis using liquid chromatography-mass-spectrometry (LC/MS/MS).

In brief, 5 µL of urine sample or EMJH medium was added to 500 µL of 1% formic acid in acetonitrile and centrifuged at 10,000× *g* for 5 min. Then, 100 µL of the supernatant was added to 300 µL of 0.1% formic acid in DDW and transferred to vials for LC/MS/MS analysis.

LC/MS/MS (6495 triple quad LC/MS; Agilent Technologies, Santa Clara, CA, USA) equipped with an InterSustainSwift C18 column (2.1 × 50 mm, φ1.9 µm, GL Sciences, Tokyo, Japan) was used to quantify DZN and IMP. In the analysis using high performance LC (HPLC), solvent A was 0.1% (*v*/*v*) formic acid and 5 mM ammonium formate in DDW, and solvent B comprised 0.1% (*v*/*v*) formic acid in acetonitrile. The following gradient was applied: *t* = 0–0.2 min, 5% solvent B; *t* = 4 min, 95% solvent B; *t* = 4–5 min, 100% solvent B. 50 °C of the column oven temperature, 0.6 mL/min of flow rate, and 5 µL of injection volume were used. Multiple-reaction monitoring (MRM) for mass spectrometry was programed as shown in Table S2. Calibration curves were applied for DZN and IMP using final concentrations of 0.5, 1, 5, 10, 25, and 50 ng/mL. The recovery rate and matrix effects were calculated using kidney and urine samples from control group mice and EMJH medium (*n* = 5), as shown in Table S3. The recovery rates showed small variations, and thus the concentrations of DZN and IMP were calculated by dividing the measured values by the recovery rate.

### 2.11. Statistical Analysis

Statistical analyses were conducted using the JMP^®^ Pro 16 software (SAS Institute, Cary, NC, USA). The Shapiro–Wilk test and Levene’s test were performed to evaluate parametric distribution and variance homogeneity of the data, respectively. The Steel–Dwass test was performed to compare the urinary shedding numbers of *L. interrogans*, the number of *L. interrogans* colonies in kidney tissues, and the expression levels of cytokine genes among each group. The Wilcoxon test was applied for the comparison of *L. interrogans* viability in in vitro assays between the control and each exposure concentration. A *p*-value < 0.05 was considered statistically significant for all analyses.

## 3. Results

### 3.1. Lethal Concentrations of DZN and IMP for L. interrogans on In Vitro Assay

**Figure 1:**
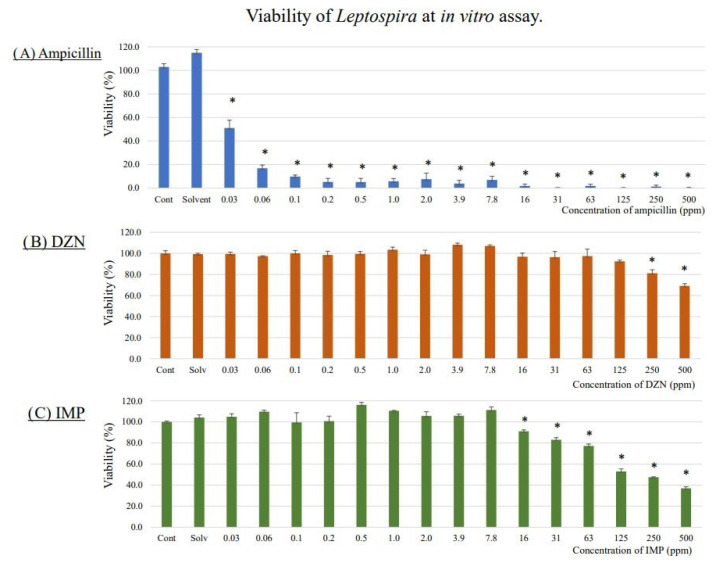
Viability of *L. interrogans* in in vitro assay using ampicillin (**A**), diazinon (**B**), and IMP (**C**) (Mean ± SE, n = 3). To begin, 9 × 10^5^ *Leptospira* cells were incubated with serial twofold dilutions of diazinon, IMP, or ampicillin ranging from 0.03 to 500 ppm (final concentration) in Ellinghausen–McCullough–Johnson–Harris medium containing 1% methanol in a 96 well plate. After 3 days of incubation at room temperature, 20 µL of 10-fold diluted AlamarBlue Cell Viability Reagent was added to each well. On the fifth day of incubation, absorbance at 570 nm was measured. The optical zero was made using the negative control well. Cell viability was expressed as a ratio with the negative control wells (%). Assays were performed in triplicate. * *p* < 0.05 (Steel test). Viability was significantly lower than control above a concentration of 250 ppm and 16 ppm for DZN and IMP, respectively.

Figure 1 shows the viability of the *L. interrogans* strain exposed to ampicillin, DZN, and IMP in vitro. In the ampicillin assay, the viability of the strain was significantly decreased when compared to the control at a concentration of 0.03 ppm (51.1 ± 6.6%, *p* = 0.007) and higher (Figure 1A). Viability was significantly lower than control above a DZN concentration of 250 ppm (81.1 ± 3.4%, *p* = 0.032) and 16 ppm for IMP (91.1 ± 1.3%, *p* = 0.041) (Figure 1B, C). DZN and IMP were stable during the assays (ratio of DZN and IMP concentrations 5 days after the assay to that before assay were 93.5–112.7% and 92.7–107.9%, respectively; Table S4).

### 3.2. Urinary Shedding of L. interrogans in Mice Exposed to DZN

**Figure 2:**
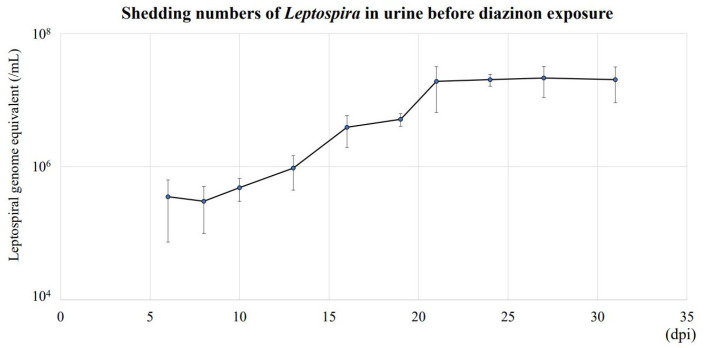
Urine *L. interrogans* quantification before diazinon exposure.

**Figure 3:**
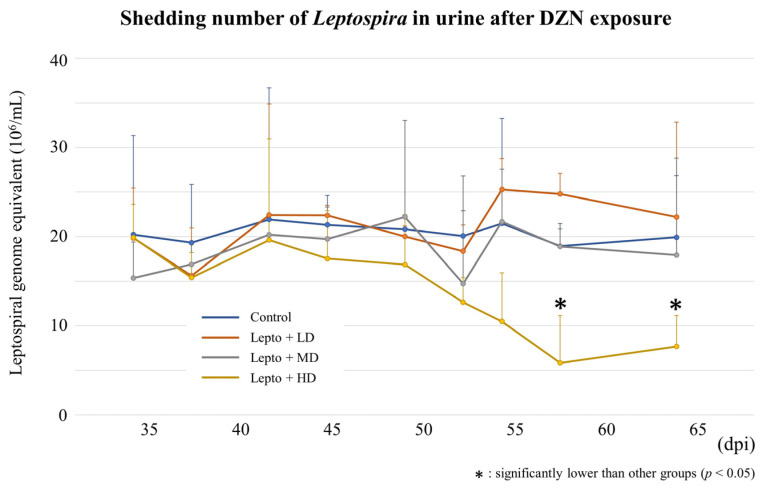
Urine *L. interrogans* quantification after diazinon exposure.

The concentration of *L. interrogans* shed in urine increased until 21 dpi and reached a plateau at approximately 2 × 10^7^ genome equivalents/mL (Figure 2). After DZN exposure, there was no significant differences in urinary *L. interrogans* among all groups until 53 dpi (Figure 3). However, urinary *L. interrogans* in Lepto + HD was significantly lower than other groups at 56 dpi (Lepto + HD: 0.58 ± 0.53 × 10^7^ /mL vs. control: 1.89 ± 0.25 × 10^7^/mL, *p* = 0.012; vs. Lepto + LD: 2.48 ± 0.23 × 10^7^/mL, *p* = 0.021; vs. Lepto + MD: 1.89 ± 0.20 × 10^7^/mL, *p* = 0.010) and at 63 dpi (Lepto + HD: 0.77 ± 0.35 × 10^7^/mL vs. control: 1.99 ± 0.69 × 10^7^/mL, *p* = 0.006; vs. Lepto + LD: 2.22 ± 1.07 × 10^7^/mL, *p* = 0.019; vs. Lepto + MD: 1.79 ± 1.09 × 10^7^/mL, *p* = 0.043). 

Urine samples of *Leptospira*-infected mice (Lepto group) were collected at 6, 8, 10, 13, 16, 18, 21, 24, 27, and 31 days post infection (dpi) before diazinon exposure. Genomic DNA was extracted from 20 µL of urine of *Leptospira*-inoculated mice. Leptospiral DNA in urine was quantified by absolute quantification of 16S rRNA gene, and the quantity of leptospiral DNA in urine was shown as leptospiral genome equivalent per mL urine (Mean ± SD, *n* = 9). The concentration of *L. interrogans* shed in urine increased until 21 dpi and reached a plateau at approximately 2 × 10^7^ genome equivalents/mL.

The dose of diazinon used was 0.2, 1.0, and 5.0 mg/kg/day for low-, medium-, and high-dose, respectively (LD, MD, HD). Continuous oral diazinon exposure via drinking water to *Leptospira*-infected mice was started at 31 dpi and continued until dpi 63. During the exposure period, urine was collected at 34, 37, 41, 45, 48, 51, 53, 56, and 63 dpi. Genomic DNA was extracted from 20 µL of urine of *Leptospira*-infected mice. Leptospiral DNA in urine of mice was quantified by absolute quantification of 16S rRNA gene. Quantity of leptospiral DNA in urine was shown as leptospiral genome equivalents per mL urine (Mean ± SD, *n* = 9). Urine L. *interrogans* in infected mice exposed to high-dose diazinon was significantly lower than other groups at 56 dpi and 63 dpi (*: *p* < 0.05, Steel–Dwass test).

### 3.3. L. interrogans in Kidney Tissues of Mice Exposed to DZN

**Figure 4:**
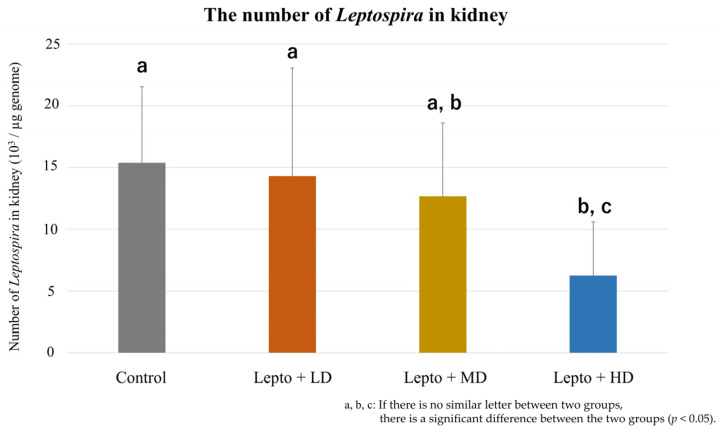
Quantification of *L. interrogans* in kidney tissues.

Kidney *L. interrogans* levels at 63 dpi (32 days after DZN exposure) are shown in Figure 4. The levels in the Lepto + HD group was significantly lower than those of the control and Lepto + LD groups (Lepto + HD: 0.62 ± 0.44 × 10^4^/µg vs. control: 1.54 ± 0.62 × 10^4^/µg, *p* = 0.019; vs. Lepto + LD: 1.43 ± 0.88 × 10^4^/µg, *p* = 0.032). There was no statistical difference between Lepto + LD and Lepto + MD groups (1.27 ± 0.59 × 10^4^ /µg, *p* = 0.33). 

Leptospiral DNA in kidney tissue was quantified by absolute quantification of 16S rRNA gene. Quantity of leptospiral DNA in kidney was shown as leptospiral genome equivalent per µg genome (Mean ± SD, *n* = 9). LD, MD, HD means low, medium, and high-dose DZN exposure, respectively. Letters above the bars (a, b, and c) represent the statistical differences. If two groups share the same letter, there is no significant difference between the two groups. If there is no similar letter between two groups, there is a significant difference between the two groups. (*p* < 0.05, Steel–Dwass test). The levels in the Lepto + HD group were significantly lower than those of the control and Lepto + LD groups.

### 3.4. Concentrations of DZN and IMP in Kidney Tissue and Urine of Exposed Mice 

The concentrations of DZN and IMP in the kidney tissue and urine of mice at 63 dpi (32 days after DZN exposure) increased depending on the orally exposed DZN dose (Table 1). Even in the HD group, DZN and IMP concentrations (2.21 ± 0.56 and 0.66 ± 0.12 ppm, respectively) in kidney tissue were much lower than the effective concentration of in vitro assays (250 and 16 ppm for DZN and IMP, respectively). In urine samples, the concentration of IMP in the HD group was near the effective concentration of the in vitro assay (12.51 ± 2.1 ppm); this was not seen for DZN (0.56 ± 0.27 ppm).

### 3.5. Pathological Characteristics of Kidney Tissues of Leptospira-Infected Mice Exposed to DZN

**Figure 5:**
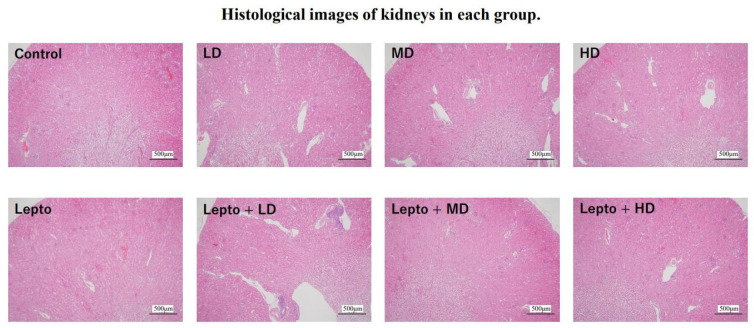
Histological examination of kidney tissue.

Figure 5 shows the histological images of kidney tissues in each group. No consistent pathological changes were observed in all groups.

Histological examination was performed using kidney tissues (*n* = 3 for each group). The paraffin-embedded kidney tissues were sectioned and stained with hematoxylin and eosin and observed using an optical microscope. LD, MD, HD means low, medium, and high-dose DZN exposure, respectively. No consistent pathological changes were observed in all groups.

### 3.6. mRNA Expression Levels of Cytokine Genes in Kidney Tissue of DZN-Exposed Mice and Leptospira-Infected Mice Exposed to DZN

**Figure 6:**
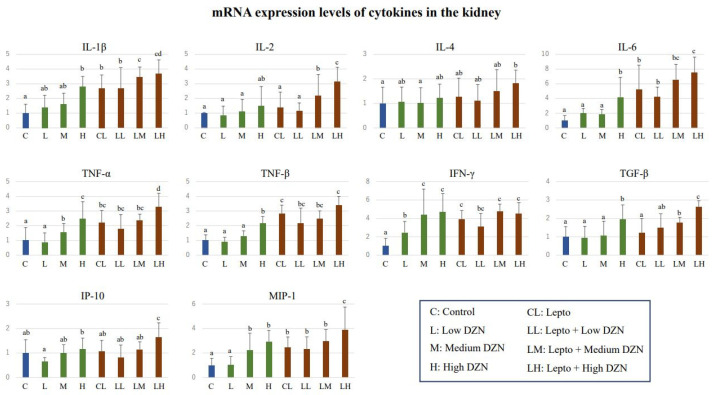
mRNA expression levels of cytokine genes in the kidney tissues of diazinon-exposed mice and *Leptospira*-infected mice exposed to diazinon. Letters above the bars (a, b, c and d) represent the statistical differences. If two groups share the same letter, there is no significant difference between the two groups.

The expression levels of cytokine genes in kidney tissue of *Leptospira*-infected mice with or without DZN are shown in Figure 6. Several cytokine gene expression levels were elevated by DZN exposure: The expression level of IFN-γ in the LD group was significantly higher than that of the control group (*p* < 0.05). Between the control and MD groups, significantly higher expression levels were observed in TNF-α, IFN-γ, and MIP-1 (*p* < 0.05). Increased cytokine gene expression was observed for IL-1β, IL-6, TNF-α, TNF-β, IFN-γ, TGF-β, and MIP-1 in the HD group when compared to the control group (*p* < 0.05). 

Elevated expression levels by *Leptospira* infection were also observed in several cytokine genes. The expression levels in the Lepto group were significantly higher than those in the control group for IL-1β, IL-6, TNF-α, TNF-β, IFN-γ, and MIP-1 (*p* < 0.05). Furthermore, DZN exposure to *Leptospira*-infected mice additively increased the expression of these cytokine genes. Compared to the Lepto group, significantly elevated gene expressions were observed in the Lepto + MD group for IL-1β and IL-2 (*p* < 0.05). The expression levels in the Lepto + HD group were significantly higher than that of the Lepto group for IL-1β, IL-2, IL-6, TNF-α, TGF-β, MIP-1, and IP-10 (*p* < 0.05). There was no significant difference in expression levels of any cytokine genes between the Lepto + LD and Lepto groups.

Total RNA was extracted from the renal cortex and used for cDNA synthesis. Absolute quantification of gene expression levels of cytokines including IL-1β, IL-2, IL-4, IL-6, TNF-α, TNF-β, IFN-γ, TGF-β, MIP-1, and IP-10 were performed (Mean ± SD, *n* = 9). Expression levels of targeted cytokine genes in each mouse group are represented as the ratio to that of the control group. Letters above the bars represent statistical differences, i.e., if two groups share the same letter, there is no significant difference between the two groups; if there is no similar letter between two groups, there is a significant difference between the two groups. (*p* < 0.05, Steel–Dwass test). Several cytokine gene expression levels were elevated as a result of DZN exposure. Elevated expression levels by *Leptospira* infection were also observed in several cytokine genes. Furthermore, DZN exposure to *Leptospira*-infected mice additively increased the expression of these cytokine genes.

## 4. Discussion

### 4.1. Effect of DZN Exposure on Leptospira Proliferation in Kidney Tissue

*Leptospira* spp. colonize and proliferate in the kidneys of reservoir animals, and some of the bacteria are excreted in the urine [2]. A positive correlation between leptospiral numbers in urine and kidney tissue was confirmed in wild rats [3]. In this study, the decreased number of *L. interrogans* in the kidney tissue following DZN exposure was likely to lead to a reduced number of bacteria shed in the urine. Although it has been reported that exposure to DZN at a concentration of 30 mg/kg caused histological changes to the kidney tissue of mice [18], this was not observed at the lower concentrations investigated in the present study. Thus, it is unlikely that *Leptospira* proliferation and/or excretion was influenced by pathological changes to renal tissue. 

In the proximal renal tubules where *Leptospira* spp. colonize, approximately 70% of filtered water by glomerulus is reabsorbed [27], and the concentrations of some chemicals increase [28]. Therefore, the concentration of IMP in the lumen of proximal tubules might be higher than that in blood and comparable with urinary concentration. *Leptospira* regularly reside in the lumen of the proximal tubules [29,30] and might be exposed to IMP in the lumen. Urinary concentrations of IMP in the present study were near the effective concentrations obtained by in vitro viability assay. Thus, this suggests that colonized *L. interrogans* in the proximal renal tubules might be exposed to the effective concentration of IMP for longer periods than the in vitro viability assay (in vitro: 5 days, in vivo: 35 days), leading to suppression of bacterial proliferation.

The expression levels of most of the cytokine genes examined increased depending on the exposure concentrations of DZN. These results are consistent with previous reports that mRNA expression levels of some cytokine genes were upregulated with DZN exposure below 6.5 mg/kg/day [13,14]. Moreover, the expression levels of cytokines, including IL-1β, IL-2, IL-6, TNF-α, TGF-β, MIP-1, and IP-10, in the kidney tissues were additively elevated by DZN exposure and *Leptospira* infection. IL-1β, IL-2, TNF-α, and MIP-1 are involved in activation of inflammatory cells, including B cells, T cells, macrophages, and natural killer cells [31,32]. IL-6 acts as a potent stimulant for B-cell proliferation and antibody production [33]. MIP-1 and IP-10 promote the migration of leukocytes [32]. The additive induction of cytokines may lead to enhanced immune system function for effective defense against *L. interrogans*. To verify this hypothesis, the interval between elevated cytokine gene levels and decreased *L. interrogans* in kidney tissue needs to be confirmed. Furthermore, it should be noted that other possible DZN-induced factors, such as oxidative stress [34] and DNA damage [13], might affect the proliferation of *L. interrogans*; this must also be explored in the future.

In this study, mice were infected with *L. interrogans* followed by DZN exposure based on vertical transmission. A significant proportion of wild rodents are infected with *Leptospira* spp. from their parents before leaving the nest [35], and wild rodents are thought to be exposed to pesticides after *Leptospira* infection. On the contrary, horizontal transmission, where rodents become infected with *Leptospira* spp. after leaving the nest, can also occur [35]. In this situation, DZN exposure can occur before mice get infected with *L. interrogans*, and DZN might affect not only the proliferation but also the initial colonization of *Leptospira* spp. in the kidneys. The effects of DZN exposure on horizontal transmission of *L. interrogans* should be investigated in the future.

### 4.2. Influence of DZN Exposure on the Infectious Risk of Leptospirosis in Human

The urine of reservoir animals is the primary infection source of *Leptospira* spp. for humans and a key factor in the environmental cycling of *Leptospira* spp. [2]. In a previous study, the infection risk of *Leptospira* spp. for humans was evaluated by applying models using parameters such as climate factor, serotypes of *Leptospira* spp., population densities, and urine volume of host animals [3]. The infection risk of leptospirosis for humans is increased as the number of *Leptospira* spp. shed in urine becomes higher [3,4]. Considering the decreased urinary shedding of *L. interrogans* due to DZN exposure in this study, it might be suggested that environmental DZN may not increase the risk of leptospirosis for people living in surrounding areas. As a limitation, it should be noted that various other chemicals that have the potential for immunotoxicities are present in the environments, but we can evaluate only diazinon-induced effects on urinary *Leptospira* shedding from this study.

## 5. Conclusions

This is the first study to examine the effects of chemical exposure on dynamics of a bacterial pathogen in reservoir animals using diazinon and *L. interrogans* as a model. Wild animals are generally important carriers of various zoonotic pathogens [36] and are exposed to a variety of environmental pollutants with immunotoxic potential, including pesticides, heavy metals, and drugs [37,38,39]. In the future, it will be necessary to investigate the effects of various environmental pollutants on infectious diseases other than leptospirosis.

## Figures and Tables

**Table 1:** Concentrations of DZN and IMP in kidney tissues and urine.

		Measured Concentration (ppm) *	
Compound	Exposure Dose	Kidney	Urine
DZN	0.2 mg/kg/day	0.25 ± 0.10	0.07 ± 0.03
	1.0 mg/kg/day	1.03 ± 0.34	0.14 ± 0.07
	5.0 mg/kg/day	2.21 ± 0.56	0.56 ± 0.27
IMP	0.2 mg/kg/day	0.23 ± 0.05	0.81 ± 0.19
	1.0 mg/kg/day	0.32 ± 0.09	2.57 ± 0.80
	5.0 mg/kg/day	0.66 ± 0.12	12.51 ± 2.1

* Average ± SD; DZN: Diazinon, IMP: isopropyl-6-methyl-4-pyrimidinol, SD: Standard deviation.

## Data Availability

Not applicable.

## Supplementary Materials

The following supporting information can be downloaded at: https://www.mdpi.com/article/10.3390/toxics11040361/s1, Table S1: Sequences of primers used for real-time PCR; Table S2: Multiple-reaction monitoring (MRM) for mass specrtrometry. Table S3: Recovery rate and matrix effect of DZN and IM|P in kidney, urine, and EMJH medium (*n* = 5), Table S4: Concentrations of DZN and IMP before and after 5 days of in vitro viability assay.
